# “Uninsurable because of a genetic test”: a qualitative study of consumer views about the use of genetic test results in Australian life insurance

**DOI:** 10.1038/s41431-024-01602-1

**Published:** 2024-04-19

**Authors:** Cassandra Muller, Lyndon Gallacher, Louise Keogh, Aideen McInerney-Leo, Tiffany Boughtwood, Penny Gleeson, Kristine Barlow-Stewart, Martin B. Delatycki, Ingrid Winship, Kristen J. Nowak, Margaret Otlowski, Paul Lacaze, Jane Tiller

**Affiliations:** 1 University of Melbourne, Parkville, VIC Australia; 2 Victorian Clinical Genetics Services, Parkville, VIC Australia; 3 Murdoch Children’s Research Institute, Parkville, VIC Australia; 4 Centre for Health Equity, Melbourne School of Population and Global Health, The University of Melbourne, Melbourne, VIC Australia; 5 Frazer Institute, The University of Queensland, Dermatology Research Centre, Brisbane, QLD Australia; 6 Australian Genomics, Melbourne, VIC Australia; 7 Deakin Law School, Melbourne, VIC Australia; 8 Northern Clinical School, Faculty of Medicine and Health, University of Sydney, Sydney, VIC Australia; 9 Department of Medicine, the University of Melbourne, Melbourne, VIC Australia; 10 Genomic Medicine and Family Cancer Clinic, Royal Melbourne Hospital, Parkville, VIC Australia; 11 Office of Population Health Genomics, Western Australia Department of Health, Perth, WA Australia; 12 Faculty of Law and Centre for Law and Genetics, University of Tasmania, Hobart, TAS Australia; 13 Public Health Genomics, School of Public Health and Preventive Medicine, Monash University, Melbourne, VIC Australia

**Keywords:** Ethics, Genetic testing, Health policy, Genetic counselling, Public health

## Abstract

Genetic testing can provide valuable information to mitigate personal disease risk, but the use of genetic results in life insurance underwriting is known to deter many consumers from pursuing genetic testing. In 2019, following Australian Federal Parliamentary Inquiry recommendations, the Financial Services Council (FSC) introduced an industry-led partial moratorium, prohibiting life insurance companies from using genetic test results for policies up to $AUD500,000. We used semi-structured interviews to explore genetic test consumers’ experiences and views about the FSC moratorium and the use of genetic results by life insurers. Individuals who participated in an online survey and agreed to be re-contacted to discuss the issue further were invited. Interviews were 20–30-min long, conducted via video conference, transcribed verbatim and analysed using inductive content analysis. Twenty-seven participants were interviewed. Despite the moratorium, concerns about genetic discrimination in life insurance were prevalent. Participants reported instances where life insurers did not consider risk mitigation when assessing risk for policies based on genetic results, contrary to legal requirements. Most participants felt that the moratorium provided inadequate protection against discrimination, and that government legislation regulating life insurers’ use of genetic results is necessary. Many participants perceived the financial limits to be inadequate, given the cost-of-living in Australia. Our findings indicate that from the perspective of participants, the moratorium has not been effective in allaying fears about genetic discrimination or ensuring adequate access to life insurance products. Concern about genetic discrimination in life insurance remains prevalent in Australia.

## Introduction

Genetic tests can provide consumers with valuable information to mitigate their risk of disease or enable early detection and/or treatment. As the utility and prevalence of genetic testing increases, ethical, legal, and social issues (ELSI) that arise with genetic testing should also be monitored. An ELSI consideration of global concern is the potential for genetic discrimination (GD). This is particularly relevant for risk-rated insurance underwriting. International research demonstrates that many consumers who have undergone or been offered genetic testing are concerned about insurance implications of genetic testing, and in some cases choose not to have testing or participate in genomic research studies [1–8].

### Use of genetic tests by Australian life insurers and concerns about genetic discrimination

In Australia, health insurance is community-rated; therefore, no individual characteristics, including genetic tests are used in underwriting [3]. However, under section 46 of the *Disability Discrimination Act* 1992 (Cth), risk-rated insurance companies (including life insurers) can legally use genetic test results to assess individual risk for insurance products, as long as they have actuarial data to justify doing so. This can lead to GD, and fear of such discrimination impacts uptake of genetic testing in Australia, both for clinical tests and research participation. One study found that when informed about potential life insurance implications, people were 50% less likely to proceed with genetic testing [9]. A recent survey of the Australian general public (*n* = 1060) found that although 92% of adults were willing to have genetic testing for medically actionable disease risk, 86% said their willingness would be negatively affected by the possibility of GD [10]. A study regarding attitudes towards genomic data sharing found that while 72% of participants were willing to donate identifiable genomic data for not-for-profit research, 83% expressed high concern about potential GD by life insurers [11].

Historical GD studies have reported numerous cases of adverse treatment of consumers by life insurers on the basis of genetic test results [12, 13]. A 2019 study by authors of this paper surveyed 174 consumers with cancer-predisposing variants and reported 49 instances of consumers with difficulties obtaining life insurance [14]. Half of those consumers had no symptoms or history of cancer and had taken proactive measures to mitigate future risk.

### Global approaches to regulating genetic discrimination

Many countries have restricted the use of genetic test results to assess individuals for risk-rated insurance products. Canada adopted the *Genetic Non-Discrimination Act* (GNA) in 2017, which prohibits the use of genetic information in services including insurance [15], with no exceptions or financial limits. In the United Kingdom (UK), since 2001 an agreement has been in place between the insurance industry and government, that prohibits the use of genetic test results in underwriting policies [16]. This applies to all results except for Huntington’s disease, for which predictive results must be disclosed to insurers for death cover policies >£500,000. All other results are protected without any financial limit. Numerous other countries, including in Asia and much of Europe, have banned or restricted the use of genetic results by insurers [2, 17–19].

The 2008 *Genetic Information Non-Discrimination Act* (GINA) in the United States of America bans the use of genetic results in health insurance. Regulation of life insurers has been approached at the state level, with Florida recently introducing a ban on use of genetic results in life insurance [20]. Public awareness of these protections has been an ongoing challenge, with studies showing poor awareness of GINA and its protections [21–23].

### The Australian Insurance Moratorium

Following an inquiry into the life insurance industry, the Australian Parliamentary Joint Committee on Corporations and Financial Services (PJC) recommended that life insurers implement a ban on using genetic results in life insurance underwriting, similar to that in the UK, and that the Australian Government consider whether legislation is required in the future [24]. The Australian Government did not respond to these recommendations. However, the Financial Services Council (FSC)—the peak body for Australian life insurance companies at the time - introduced an industry-led, self-regulated moratorium prohibiting insurance companies from requesting or using genetic test results for policies up to $AUD500,000 [25], effective from July 2019. In 2023, Australian life insurers moved from the FSC and formed the Council of Australian Life Insurers (CALI). CALI is now responsible for self-regulating the Life Insurance Code of Practice, which incorporates the moratorium initially introduced by FSC.

Concerns regarding industry self-regulation have been raised by several Australian Government inquiries in the past five years [24, 26]. We have reported elsewhere that both health professionals [27, 28] and genetic researchers [29] believe government regulation is required, and authors of this paper have called on the Australian Government to implement independent regulation on the use of genetic information [29]. Following these calls but prior to the publication of this manuscript, the Australian Government announced in November 2023 a consultation into the use of genetic test results in life insurance underwriting [30, 31]. The consultation paper included three possible policy options – do nothing; implement a total or partial legislative ban on life insurers accessing or using genetic results in underwriting; or legislate financial limits, above which life insurers will be allowed to ask for and use applicant’s genetic results. That consultation closed on 31 January 2024, and the Government is currently considering its policy response.

It is critical to monitor whether the FSC moratorium is achieving its goal of ensuring consumer access to life insurance, and meeting the aims of the PJC recommendations, including reducing the fear of GD and hesitation surrounding genetic testing. The Australian Government (via the Medical Research Future Fund) funded the Australian Genetics and Life Insurance Moratorium: Monitoring the Effectiveness and Response (A-GLIMMER) project to conduct this task from 2020–2023 [32, 33]. The present study is a part of the A-GLIMMER project and adds to the findings of an earlier paper from the A-GLIMMER project which reports findings from a survey with consumers [34]. Our research question in the present study was to explore at a deeper level the understanding, views, and experiences of consumers regarding the FSC moratorium and the use of genetic test results in life insurance underwriting. Here we report the analysis of qualitative interviews with consumers who have undergone or been offered genetic testing.

## Materials and methods

Through the broader A-GLIMMER project, an online survey (Supplementary file S1) was conducted with consumers who had taken or been offered a genetic test for disease risk, to explore their opinions and experiences about genetic testing and the FSC moratorium. The findings of that survey, spanning experiences before and after the FSC moratorium’s introduction, have been published separately [34]. To gain a more detailed and contextualised understanding of consumers’ experiences and views, we invited a sub-set of survey respondents to participate in qualitative interviews [35].

Survey respondents who had undergone predictive or diagnostic testing were eligible to participate. Predictive testing refers to testing in individuals with no known symptoms of disease; diagnostic testing takes place where symptoms are already apparent. Diagnostic testing can lead to predictive cascade testing of relatives for many conditions, and diagnostic testing is frequently the method by which at-risk families are first identified. Accordingly, the views and experiences of individuals who have undergone either predictive or diagnostic testing are directly relevant to an understanding of the impact of GD, and discrimination fears, on patients and families. People who were offered but had not yet undertaken (or had declined) genetic testing were also eligible to participate. The views of people who are deciding whether to have or have declined genetic testing are also critical to understanding the impact of GD.

To be eligible, participants had to be over the age of 18 years, live in Australia, and be able to read and speak English. Only individuals who consented to being contacted further in the online survey, and provided viable contact data, were invited to participate.

### Recruitment

Participants were initially recruited to the online survey through consumer support groups, and invitations shared by social media and email [34]. All survey participants who indicated they were willing to be contacted further were eligible for the present study. Potential participants were invited for interview via email, and purposive sampling was used to select participants whose experiences would be most relevant in answering the research question. Participants who reported experiences of discrimination since the moratorium’s introduction in July 2019 were prioritised, to ensure cases of GD, which can be difficult to capture, were explored and reported. Secondly, we prioritised participants who reported that GD considerations and/or the moratorium’s existence had influenced their decision to have genetic testing and/or to apply for life insurance (either positively or negatively). Additionally, we endeavoured to recruit participants with a range of views, to explore different perspectives. For example, despite the strong majority view on some issues in the quantitative data [34], such as the need for further regulation of the use of genetic test results by insurers, we purposely invited several participants who had expressed a contrary view.

One individual who had not completed the online survey contacted the research team independently to express interest in being included in the research. Given the aim of the qualitative study was to gather experiences and views of people who had undergone or been offered genetic testing, the study investigators checked the individual’s eligibility and sought written consent from the individual.

### Data collection and analysis

The semi-structured interview guide included topics regarding participant understanding and opinions of the moratorium, motivations for genetic testing and experiences regarding GD. The interview guides were adapted for each participant, based on their initial survey responses (see Supplementary file S2 for one participant schedule). All interviews were audio-recorded and transcribed verbatim.

#### Inductive content analysis

Inductive content analysis was performed [36], using the program NVivo (Release 1.6.2). This approach seeks to gain meaning from the dataset without a pre-established framework of categories [36].

All transcripts were thoroughly reviewed by CM for content familiarisation and to ensure accuracy between the audio recording and transcript. Transcripts were coded broadly by CM. A subset of transcripts were independently reviewed by LG, and codes were discussed between authors to ensure consistency. A coding schema was established from the first 7 transcripts (Supplementary file S3) by collating codes into preliminary content categories. The remaining transcripts were coded (and the initial 7 re-coded) to these categories (see Supplementary file S4 for a coded transcript). Once all transcripts had been coded, CM performed second-round coding to ensure no new categories were present and that saturation had been reached. From these data, the preliminary categories were discussed and refined into the final categories presented.

This study received approval from the Monash University Human Research Ethics Committee (ID 22576).

## Results

Of the 102 survey participants who consented to recontact, 39 were invited and 26 were interviewed. One additional interviewee, who met the inclusion criteria, was recruited through the recommendation of a study participant. Interviews were conducted between June and December 2022.

Total participant demographics (*n* = 27) are in Tables 1 and 2, with 52% female, and a mix of age groups, genetic testing types and associated conditions.

**Table 1:** Participant demographics.

Characteristic (*n* = 27)	*n (%)*
Sex	
Male	13 (48.1%)
Female	14 (51.9%)
Average age	48 years
Age Range	
31–40	8 (29.6%)
41–50	9 (33.3%)
51–60	6 (22.2%)
61–70	4 (14.8%)
Level of Education	
Some high school	1 (3.7%)
Grade 12/ TAFE equivalent	6 (22.2%)
Undergraduate	11 (40.7%)
Postgraduate	8 (29.6%)
Prefer not to say	1 (3.7%)
Testing type	
Predictive	9 (33.3%)
Diagnostic	16 (59.3%)
No test taken as yet	2 (7.4%)
Condition	
Hereditary Breast and Ovarian Cancer	5 (18.5%)
Lynch Syndrome	6 (22.2%)
Mitochondrial Disease	5 (18.5%)
Inherited Cardiovascular Disease	3 (11.1%)
Other	8 (29.6%)

**Table 2:** Genetic testing details of participants.

Participant (pseudonyms assigned)	Age (years)	Genetic test	Condition	Predictive/Diagnostic	Life Insurance (any form of cover)
Aaron	41	First-degree relative is positive	Lynch syndrome	Untested	Yes
Beth	38	Positive	Mitochondrial disease	Diagnostic	Yes
Chris	50	Positive	Mitochondrial disease	Diagnostic	Yes
Dani	43	Positive	Hereditary breast and ovarian cancer	Predictive	Yes
Edith	39	Negative	Adult-onset mental illness	Predictive	Yes
Frank	41	Negative	Huntington disease	Predictive	Yes
Greg	67	Positive	Inherited cardiovascular disease	Diagnostic	Yes
Harry	62	Positive	Autosomal dominant polycystic kidney disease	Diagnostic	No
Ian	53	Positive	Familial hypercholesterolaemia	Diagnostic	Yes
Jill	53	Positive	Lynch syndrome	Diagnostic	Yes
Kelly	56	Positive	Lynch syndrome	Diagnostic	Yes
Laura	61	Positive	Mitochondrial disease	Predictive	No
Madison	49	Positive	Hereditary breast and ovarian cancer	Diagnostic	No
Natalie	43	First-degree relative is positive	Porphyria	Untested	No
Oliver	45	Positive	Peutz-Jeghers syndrome	Diagnostic	Yes
Peter	38	Positive	Lynch syndrome	Predictive	Yes
Rosie	56	Negative	Hereditary breast and ovarian cancer	Diagnostic	No
Simon	37	Positive	Mitochondrial disease	Diagnostic	No
Trisha	38	Positive	Lynch syndrome	Predictive	Yes
Uma	53	Positive	Mitochondrial disease	Diagnostic	Yes
Vivienne	34	Positive	Hereditary breast and ovarian cancer	Predictive	Yes
Winona	38	Positive	Hereditary breast and ovarian cancer	Predictive	Yes
Xavier	46	Positive	Lynch syndrome	Diagnostic	Yes
Zac	45	Negative	Inherited cardiovascular disease	Diagnostic	No
Alice	71	Negative	Peutz-Jeghers syndrome	Predictive	Yes
Ben	38	Positive	Inherited cardiovascular disease	Diagnostic	No
Caleb	56	Positive	Birt-Hogg-Dubé syndrome	Diagnostic	Yes

### Inductive content analysis results

**Fig. 1:**
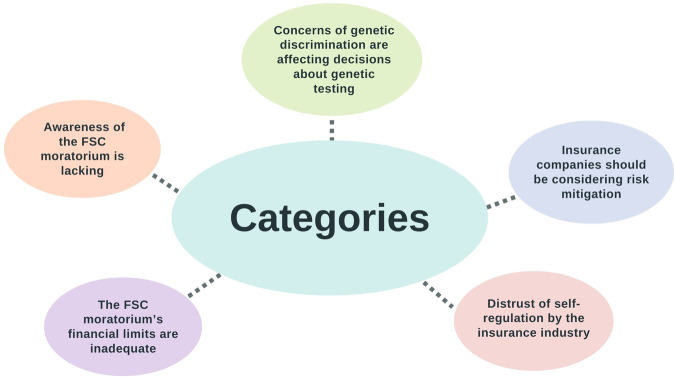
Summary of main categories identified.

Initial analysis identified a preliminary list of 16 content categories and 12 sub-categories (see Supplementary file S3). The preliminary content categories were assessed and refined into five final representative categories, (Fig. 1) with 12 corresponding sub-categories. Verbatim quotes have been selected to represent each of these categories, and additional quotes have been included in tables accompanying the text to demonstrate the breadth of responses .

**Table 3:** Themes emerging in category 1 and 2, with illustrative quotes.

Category	Subcategory	Quotes	Participant
Category ONE: Genetic discrimination concerns are affecting decisions about genetic testing	Subcategory 1.1: Impacting genetic testing decisions	Her [my sister] and I had really long talks about the implication of which one of us should get tested (..)[The geneticist] was pretty keen for us both to get tested. But we were like,“We don’t really want to run that gauntlet twice. (…)	Aaron
		I know one of my brothers hasn’t had testing done, because he didn’t want it to impact his life insurance.	Winona
	Subcategory 1.2: Impacting personal life insurance decisions	I don’t think I was ever rejected life insurance based on an application and disclosure of a genetic test because I think we kind of went there, and the advice was don’t even apply. (…) we didn’t proceed with submitting the application.	Chris
		[My sister’s] got it as well. So that’s stopped her for looking for other insurance, thinking that she wouldn’t be able to get it, would be knocked back, all of that.	Kelly
		I don’t even try to contact any life insurance for quotes or anything like that because I’m aware that they might see that on my record, and they would just say no.	Beth
	Subcategory 1.3: Impacting family testing communication	…the implications for [my children] were really profound because I had to have a really serious conversation with them that said, “Hey, I’m going to tell you I’ve been genetic tested and then we’re not going to have any other conversation about the results because there are implications for you if I tell you. I can’t actually tell you anything.	Rosie
		For me that’s my hardest thing, navigating that in the immediate term of not lying to [my daughter] and not having her worry about me, and about herself. And then my secondary concern is that, well I hope that she is negative, but that she is not penalised because of something that I unknowingly gave her.	Madison
		I’ve got a 20-year-old son and a 18-year-old daughter. They haven’t been tested yet and I’m a little bit loathe for them to do so just yet, only because it has implications on life insurance, income protection insurance, trauma, whatnot.	Caleb
Category TWO: Insurance companies should be considering risk mitigation	Subcategory 2.1: Insurers should consider risk mitigation/ management	I’m aware of it, and I’m on top of it. If I didn’t know, I probably wouldn’t be getting a screening program at my age. And at least I’m aware, so I feel that to be rejected for even having the insurance, it’s really a hard thing.	Peter
		Well in the case of it’s under control like mine was, I thought that should have been fine. But they sort of disregard the fact that these statins have solved my problem with cholesterol.	Greg
		I don’t think that I should be penalised for the fact that I have a genetic disorder, considering (…) I have a less chance of contracting breast cancer than someone in the general population. So really, I’m at an advantage compared to other people.	Winona
		If I chose not to have my genetic testing, I would then still be able to have life insurance. But I’d have to actually be at a higher risk of getting bowel cancer. To me, that doesn’t make any sense, and that’s why I feel that way. One is acknowledging the risk and then putting controls in place, and the other one is just being in denial.	Xavier
		And I think it’s all bullshit but that means because I’m on top of my health I’ve been denied, whereas someone who has a genetic condition and doesn’t know, which does happen a lot, and was not on top of regular screening, they could get life insurance.	Vivienne
	Subcategory 2.2: Insurers should not take a blanket approach to genetic results	I think they should learn to understand what the condition is, because that is the biggest issue with mitochondrial disease, nobody knows about it. They just kind of, you know, exclude it amongst everything else because it’s just too rare and too difficult to manage.(..) This is a complex condition in that you can be really severe or you can be pretty mild with your symptoms. So they should really base it on the symptoms that you declare rather than the diagnosis itself.	Beth
		There’s all these other factors that have to come into play for the disease to present even if you carry the gene for it. You can carry the gene for dementia and never end up with dementia. Do I think it’s a valid measure? No.	Rosie

### Category 1: GD concerns affect genetic testing decisions (Table 3)

Despite the FSC moratorium, participants reported that concerns about their ability to access life insurance affected genetic testing decisions (Subcategory 1.1).

“*That was really my primary concern because I’ve got two young kids now too, so I didn’t want to make myself as the primary income earner, uninsurable purely because of a genetic test*.”

[Aaron, Lynch syndrome (untested), 41y]

Some participants mentioned at-risk relatives declining genetic testing because of GD fears.

“*Even in my extended family there’s loads – there’s quite a few people who haven’t done genetic testing because they don’t want to be denied insurance cover… They’re not getting regular scans. They’re putting their health on the back foot because of all this. This is ridiculous*.”

[Vivienne, Hereditary breast and ovarian cancer (positive test result), 34y]

Some participants also reported GD concerns affected their decisions around life insurance applications - some reported not bothering to apply for life insurance policies, because they believed or had been advised that they would be unsuccessful (Subcategory 1.2).

Several participants reported concerns about their genetic results affecting their children’s future access to life insurance directly impacting family communication about testing (Subcategory 3).

“*Both of my girls now are probably at the age where they should be tested, but I’m not letting them get tested until they start working and they’re able to pay for life insurance*.”

[Jill, Lynch syndrome (positive test result), 53y]

### Category 2: Consideration of risk mitigation/management (Table 3)

Participants commonly described risk mitigation as a key reason for having genetic testing. Many were concerned that insurance companies were using genetic test results to underwrite policies, but not considering management and mitigation (Subcategory 2.1).

“*My lifestyle, with my medication, quite possibly puts me at a lower risk level than what some other person who doesn’t take reasonable care of their health… basing your risk just on your condition and not on how it’s being managed is wrong*.”

[Ian, Familial hypercholesterolaemia (positive test result), 53y]

Many participants also mentioned concerns with insurers taking a “blanket approach” to declining cover or penalising applicants with genetic test results. (Subcategory 2.2).

“*I felt that it was pretty unfair to take this blanket approach … based on the limited knowledge of those genetics at the time. And I would imagine the even more limited knowledge of those genetics in the underwriters*.”

[Chris, Mitochondrial disease (positive test result), 50y]

Some participants, who held a minority view, stated that insurance companies should be able to consider genetic tests in their underwriting, though there was still an expectation that risk-reducing measures would be taken into account as well .

“*I don’t think there should be any exceptions… I dare say most people with a genetic disease it’s not their fault, but we live in a commercial world*.”

[Harry, Autosomal dominant polycystic kidney disease (positive test result), 62y]

“*I know it’s controversial, but I think – because a life insurer’s job is to assess risk, and I think that [genetic results] is key information to doing that. And because there are things that you can do for most things to reduce your risk, I think that it would only be fair that life insurers also take into account the measures that you’re taking to reduce your risk. But I get it. It’s hard, you get discriminated against. But that’s essentially what pricing for life insurance is*."

[Trisha, Lynch syndrome (positive test result), 38y]

**Table 4:** Themes emerging in categories 3, 4 and 5, with illustrative quotes.

Category	Subcategory	Quotes	Participant
CATEGORY THREE: Distrust of the insurance industry and self-regulation	Subcategory 3.1: Poor history of industry-led agreements	[Self regulation] has a bad history of not being done correctly and self-regulation leaves a lot to be – you can regulate as hard or as easy as you like, put it that way. Who’s holding you accountable? Yourself?	Oliver
	Subcategory 3.2: Financial motivations	The government has to legislate. It’s the only way that we can safeguard people like my son and others. If we don’t have legislation, it’s just open too much to a market force of the industry, and the industry is extremely profit orientated.	Alice
	Subcategory 3.3: Need for certainty	It’s very subjective if the actual insurance industry itself, like you know if you’ve got self-regulation, it’s not, that can change at anytime.	Kelly
		I don’t think they should be allowed to arbitrarily just make their own decisions because things may not be going their way necessarily. I just do think there needs to be some regulation there at a statutory level so there is clear oversight for all parties and the customers/clients can see what their rights are and know what they can expect.	Caleb
CATEGORY FOUR: The moratorium’s financial limits are inadequate	Subcategory 4.1: Inadequate to cover families' needs	I think it’s to do with the cost of living to be honest and house prices... if you’ve got 500,000 that’s not going to pay the mortgage off necessarily, and it’s things like that that you need when someone, a loved one especially if they’re a breadwinner passes away.	Uma
	Subcategory 4.2: There should be no limits	I don’t think there should be a financial limit. I think that that is just creating another negative for anyone that has a genetic disorder. And once again, in my case, I don’t think that it’s actually going to have a negative impact on my health at all, so I don’t think it should have a cap	Winona
		Why should my parents be penalised if their coverage is a million dollars, versus $500,000, if the gene hasn’t been activated, right? I probably think that just in general, probably genetics shouldn’t come into it.	Ben
CATEGORY FIVE: Awareness of the FSC moratorium is lacking	Subcategory 5.1: Lack of awareness impacts effectiveness	I’m not convinced about that FSC moratorium’s effectiveness anyway because while it may exist on paper somewhere, I’m not sure the insurers and the people who actually do the insurance stuff even know about it.	Aaron
		I’m not even sure if [genetics service] know about it, because that’s who we go and see, that there is a FSC moratorium… And also the fact that I didn’t know anything about it. So they can have this information in place, but have kept it quite quiet and nobody even knows about it.	Kelly
	Subcategory 5.2: Awareness comes with privilege	My genetic testing was kind of – I’ll be really blunt and use the word ‘privileged’ because I could afford to essentially put $1000 down between paying the gap to see the private geneticist... And I think that’s probably why I knew a fair bit more about the FSC moratorium because I’d seen a private geneticist with a two-hour appointment.	Rosie

### Category 3: Distrust of insurance industry and self-regulation (Table 4)

Many participants expressed distrust of the insurance industry, with several noting the poor history of self-regulation (Subcategory 3.1).

“*Any industry-led model has never been particularly successful, and even more so in the human or the financial services in the world… I do think it should be in legislation; at the very least it should be in an act of parliament that declares the sanctity of the genetic information*.”

[Rosie, Hereditary breast and ovarian cancer (negative test result), 56y]

“*There’s so many examples of industry led agreements that just aren’t worth the paper they’re written on*.”

[Natalie, Porphyria (untested), 43y]

Some participants expressed concern that the insurance industry is driven solely by financial motivations (Subcategory 3.2).

“*I have no confidence whatsoever in the insurance industry to manage themselves because I don’t believe their interest are with me as the individual. (…) They’re with their shareholders. That’s why government should bloody mandate it*.”

[Aaron, Lynch syndrome (untested), 41y]

“*The Government you would hope would have the greater good in mind and the public good rather than from the point of view of a company with shareholders*.”

[Frank, Huntington’s disease (negative test result), 41y]

Participants shared concerns about lack of certainty around the FSC moratorium continuing in the future. (Subcategory 3.3).

“*If it’s not really legislated, then they can just pull out of it at any point in time. I don’t trust them to honour it*.”

[Jill, Lynch syndrome (positive test result), 53y]

One participant, however, did not believe government regulation is needed, stating that insurers should be able to ask for what information they need to be aware of the risk.

“*From a commercial point of view they should be able to make that decision, but that’s their decision rather than a government saying, “you must do this, you must provide all those details*”

[Harry, Autosomal dominant polycystic kidney disease (positive test result), 62y]

### Category 4: Financial limits are inadequate (Table 4)

Many participants mentioned that the moratorium’s financial limits are not high enough to cover their financial needs. Participants were worried that the current limits of $AUD500,000 for life insurance and $AUD4000/month for income protection would be insufficient to adequately care for their families’ needs (Subcategory 4.1).

“*The dollar values need to reflect true dollar costs of the current Australian cost of living... I don’t know any of us that have got less than a million dollars’ worth of life insurance these days. $500,000 is two-fifths of stuff all*.”

[Rosie, Hereditary breast and ovarian cancer (negative test result), 56y]

“*I’d just still leave my family with a debt. (…)I think that’s the token effort is we’ll give you 500,000 (…) Maybe 500,000 10 years ago was good, but [now] it doesn’t buy you shit*.”

[Oliver, Peutz-Jeghers syndrome (positive test result), 45y]

Some participants believed that there should be no financial limits at all to the protection offered (Subcategory 4.2). While most participants felt the financial limits were inadequate, a few participants noted that financial limits are a way for insurance companies to remain sustainable, and that *‘having some cover is better than no cover’* [Frank, Huntington disease (negative test result), 41y].

### Category 5: Awareness of the FSC moratorium is lacking (Table 4)

Many participants reported that they were not previously aware of the FSC moratorium. The lack of awareness raised concerns around its effectiveness, some stating that they were not sure insurers or even genetic health professionals were aware of the agreement (Subcategory 5.1).

Some participants also pointed out that there is socioeconomic privilege in being aware of the FSC moratorium, and expressed concern for those without their resources or education (Subcategory 5.2).

“*Others, that may not have the resources or even the level of education that maybe our family have, that don’t quite understand the nuances and the terminology and the wording and that kind of thing, that it can be quite biased towards the insurance companies*.”

[Laura, Mitochondrial disease (positive test result), 61y]

This was echoed by another participant, who upon learning about the moratorium, wrote to her insurance company to dispute the exclusions put on her policy.

“*It’s [the moratorium] made a difference in my life, so I’m very thankful for that. But maybe the key is just to get it out there, to make sure that people are aware that they have those rights. Because if I didn’t look it up and then kind of question them, I would have probably just accepted [the exclusions]*”

[Dani, Hereditary breast and ovarian cancer (positive test result), 43y]

## Discussion

This study explored the views of people who have had or been offered genetic testing for disease risk on the FSC moratorium and their experiences with GD. Our results show low trust in the life insurance industry’s self-regulation, and strong support for legislation from the Australian Government. Even after the introduction of the moratorium, these consumers remained concerned about the implications of their genetic test results on life insurance, and these concerns affected decision-making about genetic testing. Participants also showed little knowledge of the moratorium, or were concerned about others not being informed about it.

### Distrust of self-regulation

We have previously reported concerns about industry self-regulation of insurer use of genetic tests [27, 28, 37] and similar findings are reported here. Concerns about industry self-regulation have also been expressed through formal government inquiries, including the 2019 Australian Royal Commission into Misconduct in Banking, Superannuation and Financial Services Industry, which found that insurance companies were not always acting in utmost good faith, and misconduct occurred without wrongdoers being held accountable [26].

Participants in our study raised concerns that the self-regulated nature of the moratorium means the agreement could be revoked at any time, and that insurers are inherently focussed on their own financial interests rather than those of consumers, making self-regulation inappropriate. These views are consistent with the views of health professionals [27, 28] and researchers [29] previously surveyed through the A-GLIMMER study, who expressed strong majority views that government legislation is required on this issue.

### Impact of discrimination concerns

The FSC moratorium acknowledges the concern of GD in life insurance and the importance of not dissuading the public from genetic testing [25]. However, our study provides concerning evidence that some participants and their at-risk family members continue to decline genetic testing because of GD fears. One participant’s reports of intentionally dissuading their children from testing because of potential insurance implications, despite awareness of the benefits of testing, are concerning.

Most participants in our study had already had genetic testing. This suggests the value they saw in genetic testing may have outweighed GD concerns for themselves, though many remained concerned about GD implications. Importantly, this suggests consumers may consent to genetic testing *despite* having significant fears of insurance implications, because of the health risks they face in not having testing. Similar findings arose from the A-GLIMMER study with genetic researchers, who reported that many individuals are afraid of insurance implications but continue with genetic testing despite them, if the need for a diagnosis outweighs the financial concerns [29]. This is consistent with international findings that significant proportions of people who had proceeded to have genetic testing did so despite being worried about the insurance implications of having the test [5–7].

### Consideration of risk mitigation

Genetic testing for medically actionable diseases can allow for risk mitigation in the form of lifestyle, screening and/or preventive measures. Some individuals who have had predictive testing and can proactively manage their risks may be in a better preventative health position than those who decline testing. For example, familial hypercholesterolaemia (FH) is the most common cause of premature cardiovascular disease, but is largely undiagnosed in the general population [38]. When FH is well-managed, the overall risk of myocardial infarction can be reduced by 76%, similar to the general population [39]. Similarly, risk reduction surgery for individuals with a *BRCA1/2* pathogenic variant can reduce breast and/or ovarian cancer risk to below general population levels [40].

The FSC moratorium states that insurance companies consider evidence-based treatment or preventative measures when underwriting policies [25]. The *Disability Discrimination Act 1992* (Cth), also requires that insurance companies only discriminate on actuarial grounds, meaning all evidence of risk reduction must be considered. Our study showed low awareness of insurers’ obligation to consider risk management. Additionally, an earlier study found that only 15% of Australians knew how to lodge an official complaint if they felt they had been mistreated based on their genetics [12]. The poor transparency in insurers’ decision-making [37], and limited recourse to appeals for affected consumers [14, 41], further limit the efficacy of the FSC moratorium’s efficacy in providing consumer protection.

### Inadequacy of financial limits

Concerns about the financial limits of the FSC moratorium’s protection confirm our previous findings in which health professionals reported that the financial limits were too low to adequately protect their patients’ financial needs [27, 28]. Researchers and financial advisers have similarly raised concerns about the low financial limits [29]. Arguably, the financial limits set by the FSC moratorium do not reflect Australia’s current cost of living. As of May 2022, the average weekly earnings in Australia were $AUD1,769.80 [42] and the average home loan was $AUD609,000 [43], both significantly higher than the FSC moratorium’s current limits. The Australian economic climate in the 12 months following has seen significant inflation, interest rate rises and cost of living pressures, further increasing the amount of insurance coverage required. Several participants in our study noted that they would require well over $AUD1million of coverage to be adequately covered.

Some participants considered that financial limits may be necessary for the industry’s sustainability. However, no substantial evidence suggests this will be the case [44]. Canada has successfully banned the use of genetic test results in underwriting insurance without financial limits [45]. Substantial modelling commissioned by the Office of the Privacy Commissioner in Canada indicated that a complete ban would have negligible impact on insurers [46–48]. The UK Code [45] does not appear to have caused instability in the insurance market [44].

### Poor awareness of the moratorium

Participants had limited awareness of the FSC moratorium. Consumers’ understanding of the life insurance implications of genetic testing is vital for individuals to make informed testing decisions. Further, as discussed above, awareness of the guidelines is necessary for protection of their rights in the process of gaining insurance.

Strengths of this study include structured interviews with consumers with a wide variety of experiences and genetic backgrounds, allowing for meaningful insight into consumer views about GD. We acknowledge the potential for response and selection bias in our study, as those who feel the most strongly about these issues may have responded to the original online survey and in turn been selected for this study. We purposively sampled several individuals with minority views to ensure they were captured in our qualitative analysis.

The deterrent effect of insurance fears on genetic testing has been demonstrated [9, 49, 50], but it is challenging to engage individuals who have declined genetic testing in research to explore their reasons. While we have reported some findings about such individuals, our study similarly struggled to recruit testing decliners. Further targeted research is required to reach individuals who have declined testing for insurance reasons and gain a deeper understanding of their decision-making.

Overall, our findings suggest that the FSC moratorium has not assuaged consumer fears regarding the risk of GD or access to life insurance. Improved access would require adequate protections to meet consumer needs, consumer awareness, and confidence that insurers would comply. Unfortunately, consumers continue to have concerns across these areas. Participants stated a strong preference for government regulation to ensure adequate protection against GD in life insurance and provide certainty to individuals considering genetic testing. The Australian Government is now considering its policy response to its public consultation into the use of genetic results in life insurance underwriting.

### Supplementary information

Supplementary file S1

Supplementary file S2

Supplementary file S3

Supplementary file S4

## Data Availability

Numerous data are made available via supplementary materials. Additional data can be made available on reasonable request.
