# Supplementary material for: “Uninsurable because of a genetic test”: a qualitative study of consumer views about the use of genetic test results in Australian life insurance
Source: Eur J Hum Genet. 2024 Apr 19;32(7):827–36. doi: 10.1038/s41431-024-01602-1 (PMC11219861; doi:10.1038/s41431-024-01602-1)
Supplement: Supplementary file 2 — Supplementary file S2 [file 41431_2024_1602_MOESM2_ESM.pdf]

Interview Schedule 272

This participant has had genetic testing for BRACA 2 and received a positive result. They have not had a first-degree relative be diagnosed with an associated cancer. They have had risk-reducing surgeries including a bilateral mastectomy and a hysterectomy. The participant also undertakes regular screening. She applied for Life Insurance, TPD, Income Protection and Trauma/critical illness cover after the moratorium was introduced and had conditions placed on the cover. Her insurance does not exceed the limits of the moratorium. The insurer told her that she would not be covered for certain things - such as any cancers relating to the gene.

This interview will be recorded, and your answers may be published but will be de-identified. Are you happy to proceed?

As you know, you recently completed a survey about genetic testing and the life insurance moratorium. I have your answers in front of me, and I will be asking questions that expand on your responses to that survey.

Topic	Main Questions	Prompts
**Background/Introduction**<br>*Keep this short, predominately use as warm up questions*	This study is asking about experiences with genetic testing and specifically considerations around life insurance. To start with, could you tell me a bit about what led you to seek genetic testing?	• What were you hoping to learn from the genetic test?<br>• Did you have any concerns at the time about genetic testing?
	You indicated on the survey that you were unaware of the Genetics and Life Insurance Moratorium. Can you tell me how much you now know about it?	• How did you become aware of it? What prompted you to find out about it?<br>• In your own words, what does it mean?<br>• Are there aspects of it that are unclear to you?
**Personal Experience – Decision Making**<br>*These are the most important questions*	I can see that you have basic cover for Life Insurance and Trauma/Critical illness cover through your superannuation. Can you tell me about your experience obtaining this cover?	
	You told us that there were conditions placed on your policies. Can you tell me more about that?	• What did the insurer tell you?<br>• How has this affected you?<br>• Is there anything that you think needs to be changed?

	Is there anything else that you think I should know about this experience?	
**General opinion of the moratorium**<br>*These questions are not as important but can be used for further warm up questions participant is reluctant*	You indicated that you believe life insurance companies should not be allowed to use applicants' genetic test results to decline an application, restrict cover or increase the cost of premiums. Can you elaborate on this?	• Do you think this applies in all circumstances or might there be times when it is appropriate for insurance companies to use the results?
	You indicated that you strongly agree that the Australian government should introduce legislation (which is made and enforced by the government) to regulate life insurers' use of genetic test results. You also told us that it was a negative aspect of the moratorium that it is not law. Could you speak about why you feel this way?	
	On the survey, you told us that you were unsure if insurance companies should be able to ask for genetic test results and later indicated that it is negative that the agreement only applies to cover up to certain amounts ($500,000 for life cover). Can you tell me about your thought process?	
	Is there anything else you would like to tell me?	

Close: Thank you for your time today. I will be going away and making notes from what you've told me. If I have need something clarified, or a little more detail, would you be happy for me to contact you again?
