# Supplementary material for: “Uninsurable because of a genetic test”: a qualitative study of consumer views about the use of genetic test results in Australian life insurance
Source: Eur J Hum Genet. 2024 Apr 19;32(7):827–36. doi: 10.1038/s41431-024-01602-1 (PMC11219861; doi:10.1038/s41431-024-01602-1)
Supplement: Supplementary file 3 — Supplementary file S3 [file 41431_2024_1602_MOESM3_ESM.pdf]

NVIVO- Consumer Perspectives on the Australian Genetics and Life Insurance Moratorium CODES

Codes

Name
Added stress
Challenging insurers and using genetic test results to lower risk
Fear of GD is impacting family communication
Fear of genetic testing
No impact
Financial limits are not high enough
Government regulation is a must
Bad history
No gov reg
No trust
Permanency
I'd be happy to pay more if I was a higher risk as long as it's reasonable.
It's different in other countries
Moratorium awareness is lacking
Privilege
Not enough science

Name
Putting off insurance
Putting off testing
No effect
Reason for testing
Children
Family history
Other family member was diagnosed
Risk management
Symptoms
Shouldn't use testing at all
Still feeling discriminated
Where do you draw the line?
