# Supplementary material for: “Uninsurable because of a genetic test”: a qualitative study of consumer views about the use of genetic test results in Australian life insurance
Source: Eur J Hum Genet. 2024 Apr 19;32(7):827–36. doi: 10.1038/s41431-024-01602-1 (PMC11219861; doi:10.1038/s41431-024-01602-1)
Supplement: Supplementary file 4 — Supplementary file S4 [file 41431_2024_1602_MOESM4_ESM.pdf]

Name: audio133 (1)

Created on: 14/08/2022 12:30:04 PM
Created by: CM
Modified on: 18/09/2022 5:58:04 PM
Modified by: CM
Size: 76 KB

¶1:

¶2: A: Hi Cassandra, how are you going?

¶3: **Q:** Hi. Good. How are you?

¶4: **A:** Good thank you. You can hear me okay?

¶5: **Q:** Yeah. Let me know if I go bad though, because my – I've had to connect to my phone today, because my Internet's not working.

¶6: **A:** Okay.

¶7: **Q:** If it gets too bad, I'll switch to my actual phone and get off my computer.

¶8: **A:** Okay. Yep. No drama at all.

¶9: **Q:** All right. Thank you. Thanks so much for being here.

¶10: **A:** No, no problem at all.

¶11: **Q:** All right. Cool. I'm just going to let you know straight up, I've got my other monitor here, which has all my questions I'm going to ask you, as well as your survey answers. If I'm looking over here, that's why.

¶12: **A:** I'm looking at the screen here so – I'm going back to my old original answer which I can't remember when it was, 2021, so yeah, I might be referring back and forth.

¶13: **Q:** No, that's okay. If your answers have changed it doesn't matter. Just say what you think. Okay. I'll just let you know that we are recording at the moment and your answers may be published but they'll be de-identified.

¶14: **A:** Yep.

¶15: **Q:** Are you happy to proceed?

¶16: **A:** Yep, no problem.

¶17: **Q:** Yep. Okay. Cool. As you know you recently completed a survey about genetic testing and life insurance moratorium. I have your answers in front of me and I'll be asking questions that expand on your responses from the survey. In particular, we're really

- Uncertain of getting cover
- Need a guarntee
- Needs to be legislated
- Hope that the moratroium will help others
- Insurers being unaware of the condition
- Many people do not have the privilege to be aware of their rights
- Genetic testing can break cycle of disease
- GD in insurance is not really talked about
- Informed consent is important
- Word of mouth
- A shock to get knocked back
- Finanical limits
- Insurance rejection adds stress to the process
- Impact of Moratorium
- You don't know where the trigger point where I can't get insurance anymore
- Some cover is better than no cover
- I felt that the genetic counselling was pure science.
- Awareness of potential GD
- I don't have disease. It's pretty cut and dry"
- Awareness through support groups
- Using genetic test results
- Challenging the insurance

Coding Density

interested in getting to know about your experiences with genetic testing in relation to life insurance and your opinions on the current moratorium. Does that sound all right?

¶18: **A:** Yep, it sounds good, yep.

¶19: **Q:** Just to start with, could you just tell me a little bit about your personal experience with genetic testing and what led you to get tested?

¶20: **A:** Yep. My father had Huntington’s disease and I can’t remember what it was but a number of years ago I was getting at the stage where I was married for a few years, we were thinking about having kids and so I went down the path for genetic testing. I did that at the Murdoch by the VCGS in Melbourne. Before I went through that testing, and the test ended up – it was negative, but before I went on this journey I contacted Huntington’s Victoria who were quite supportive in just giving the background about what things to consider, insurance, other sort of things like that, who to tell, when not to tell and this sort of stuff at work and so on. They put me in touch with a guy called Felix Solomon I think his name is from MLC who was working pro bono for Huntington’s Victoria giving financial advice. He was the one who guided me through the insurance side of things and the financial side of things before getting the testing. That was my sort of background of getting it all – trying to get it started. I got my, I think it was TPD and life insurance sort of – I boosted that up pretty high before I did any sort of testing but I remember there was a question I had to get the GP to fill out. I think it was Uni Super I was with and underwritten by TAL, but I had to write it on the form. There was a specific question about Huntington’s disease, “Do you have that in your family? Is that” – and so that was the case. I got a 200% loading on, I can’t remember what it was, whether it was the TPD or one of the other parts of the insurance, but that was a non-negotiable [dossier] 00:04:11 200% loading on that because of the risk. I went through that -

¶21: **Q:** Massive.

¶22: **A:** Yeah [common sense] 00:04:18 I was like okay, just pay it, whatever, pay it. And then went through the testing. Testing was negative. As soon as I got the test I contacted Uni Super or whoever the people was, I can’t remember whether it was TAL or Uni Super, I said, “Please remove the loading. It’s now officially I don’t have this.” And then they asked me to provide evidence or something that I don’t have any comorbidities and they want to do another assessment on whether the condition still could be impacting me or something, but I said, “I don’t have disease. It’s pretty cut and dry”, and they’re like, “Well, we can’t assess this until you have – you do this full report again, all your habits and everything, a full policy again.” I’m like, “Just remove the loading. I’ve already done the policy. You put the premium on because of that particular aspect so just remove that and we’re all good.” They’re like, “No, we want to have – do a full medical again on you.” I’m like, “But that’s not the point of why you put the loading on. It wasn’t because of anything else. It was just because of that so just remove it. It's easy.”

¶23: **Q:** It wasn’t because of your habits or anything, it was just -

¶24: **A:** No.

- Uncertain of getting cover
- Need a guarntee
- Needs to be legislated
- Hope that the moratroium will help others
- Insurers being unaware of the condition
- Many people do not have the privilege to be aware of their rights
- Genetic testing can break cycle of disease
- GD in insurance is not really talked about
- Informed consent is important
- Word of mouth
- A shock to get knocked back
- Finanical limits
- Insurance rejection adds stress to the process
- Impact of Moratorium
- You don't know where the trigger point where I can't get insurance anymore
- Some cover is better than no cover
- I felt that the genetic counselling was pure science.
- Awareness of potential GD
- I don't have disease. It's pretty cut and dry"
- Awareness through support groups
- Using genetic test results
- Challenging the insurance

Coding Density

¶25: **Q:** - your family history, that's all. Yeah.

¶26: **A:** That's none of their business at that point. I've already got the premium – the policy. Yeah. And then I was like, "Well, you don't go back to other people halfway through their policy and get them to redo their applications or anything like that if their circumstances have changed. My circumstances haven't changed. They've always been the same. I just now know that I don't have this, so just take off the loading. It's easy." And then they wouldn't. I said, "Fine. Okay. Let's go." So we went to – I think it was the superannuation tribunal, whatever it was, got all that way and they just said, "Look, we have to assess you as a new policy", and they just didn't get it. They really didn't get it. It was just so stressful and a waste of everyone's time. And then – and the lawyer – there was like four lawyers in the room or something, it was on a phone call but it was like God, it was just me representing myself and them not understanding and them admitting, "Look, we don't know anything about this condition. We'll have to make sure we" – it's like, "You've written it on the paper as a distinct thing. Educate yourselves about it." Anyway, they said they would go back and they would re-think about their process and basically they repaid from when I did tell them that I was gene negative they backdated the payment. It was only – it wasn't much but from that point when I did start the complaint process – or not the complaint process but just inform them that I was gene negative they backdated that. That was the settlement I guess you could call it which was a couple of hundred bucks I think but -

¶27: **Q:** Yep.

¶28: **A:** It wasn't a great experience. It took many many years.

¶29: **Q:** Oh okay, years. Yeah, just to backtrack, you've given a great overview so sorry if things get a bit repetitive because I'll probably ask you more specific questions, but just to contextualise this can you tell me when was this happening?

¶30: **A:** I can't recall. I did send the document to Jane I think.

¶31: **Q:** Oh cool. Yep.

¶32: **A:** She's got a full timeline and their lawyers or whatever, did it quite nicely.

¶33: **Q:** Perfect. Yep.

¶34: **A:** So the full timeline or what I did when and exact dates of everything but took years to get through the tribunal because you have to go back and go through the complaints process at TAL, then they say no, and then go back and forward and – so I withdrew the complaint. The outcome was they wanted me to withdraw the complaint, do a new medical and then they would backdate the payment and I was like, "Okay, fine. As long as you – [unclear] 00:08:13 you were going to pay I'm happy with that." Maybe reflect on just how complicated it is for people to go through.

¶35: **Q:** Yeah.

- Uncertain of getting cover
- Need a guarntee
- Needs to be legislated
- Hope that the moratroium will help others
- Insurers being unaware of the condition
- Many people do not have the privilege to be aware of their rights
- Genetic testing can break cycle of disease
- GD in insurance is not really talked about
- Informed consent is important
- Word of mouth
- A shock to get knocked back
- Finanical limits
- Insurance rejection adds stress to the process
- Impact of Moratorium
- You don't know where the trigger point where I can't get insurance anymore
- Some cover is better than no cover
- I felt that the genetic counselling was pure science.
- Awareness of potential GD
- I don't have disease. It's pretty cut and dry"
- Awareness through support groups
- Using genetic test results
- Challenging the insurance

Coding Density

¶36: **A:** Anyway.

¶37: **Q:** Yeah. Okay. Thank you. We'll go into a bit – some specific questions. But what you were saying then as well, you said – the information about life insurance that you got and that sort of stuff when you were going for this test, it was from the support group, was it?

¶38: **A:** Yep. Yeah. I knew speaking to – because Huntington's, it's a family thing so people have generally been through it before in family but we were kind of the first in our family to go through it, through my dad, but no one really talked about the insurance stuff but it was kind of like just be careful with insurance and things like that because if you get tested without getting that sorted you may not be able to get more – increase your policy or whatever. I had that in back of my mind but it was really through the support group that gave the official details and I've then passed it on to my cousins and so on who are going through the same sort of thing, "Get that sorted. Go and speak to them. Here's the guy's name. Just get it sorted." But it's only like word of mouth. I mean, no one really – I don't know, it's just from, yeah, family member to family member really.

¶39: **Q:** Yeah. Okay. Interesting, yeah, that it has to be kind of through people unrelated to either genetic tests or the life insurance that were telling you what to do.

¶40: **A:** Yeah. I mean, when you fill out the policy it says straight up if you've got a family history of Huntington's yes or no, and that just disqualifies you straightaway, or not disqualifies you but adds this extra layer, 200%.

¶41: **Q:** Yep. Okay. On the survey I think at the start of it, you'd said you hadn't heard of the genetics and life insurance moratorium. Can you just tell me in your own words how much you know about it now?

¶42: **A:** I was going to ask you – I don't know that much, to be honest.

¶43: **Q:** That's okay. That's fine.

¶44: **A:** Yeah, I really don't know that much about it but if you're able to give a background at all. I don't know if you need to but I don't know that much about it. I haven't really – I kind of feel like I've moved -

¶45: **Q:** On?

¶46: **A:** - on a little bit. Yeah. I haven't really invested my time necessarily in it. But I'm very interested in it. I mean, it impacts my family members, so, yeah, for sure.

¶47: **Q:** Yeah. No, that's okay. We just like – part of this research anyway is just knowing how much people know about it especially people who it has a direct impact on, so if you don't know much that's fine. I don't know a lot either. Yeah. Okay.

¶48: **A:** I think I have at certain points read up about it and then it's kind of gone in and out and then I've kind of read up again and then I'm like I can't remember what is it about but -

- Uncertain of getting cover
- Need a guarntee
- Needs to be legislated
- Hope that the moratroium will help others
- Insurers being unaware of the condition
- Many people do not have the privilege to be aware of their rights
- Genetic testing can break cycle of disease
  - GD in insurance is not really talked about
- Informed consent is important
  - Word of mouth
- A shock to get knocked back
- Finanical limits
- Insurance rejection adds stress to the process
- Impact of Moratorium
- You don't know where the trigger point where I can't get insurance anymore
- Some cover is better than no cover
- I felt that the genetic counselling was pure science.
- Awareness of potential GD
- I don't have disease. It's pretty cut and dry"
  - Awareness through support groups
- Using genetic test results
- Challenging the insurance

Coding Density

¶49: **Q:** Yeah. No, that's okay. So basically it's just an agreement between – it's managed by life insurance companies themselves to say that they're not going to ask for genetic test results up to a certain amount of money. I think it's 500,00 for just general life insurance that they can't ask for genetic test results for, but it also means that if you do get a positive – a negative sorry, that negates family history and you can tell them about that as well and they can't use that family history.

¶50: **A:** Yep, yep, yep.

¶51: **Q:** Yeah. Okay. D you've already done this, I'm just going to ask you to cut your story down a little bit into a chunk and just can you tell us a little bit about when you applied for your life insurance total permanent disability and IP, and you were denied one cover and that the death cover had a 200% loading, is that right?

¶52: **A:** I believe that's correct, yes.

¶53: **Q:** Okay. I asked do you remember when you've applied for it and you're not sure?

¶54: **A:** It says before 2019. Let me just check that dossier. Sent to Jane Tiller, is that her name?

¶55: **Q:** Yeah.

¶56: **A:** Jane Tiller. I should have been more prepared. It was before 2016. I applied for death cover, income protection and TPD. I was approved for death cover with 200% loading and denied cover for TPD and income protection.

¶57: **Q:** Yep. Okay. Can you tell me a little bit about that, what that process was like?

¶58: **A:** It was pretty straightforward really. I mean, it was just filling out the forms and because I had that question of – on Huntington's and I couldn't – when my dad had been diagnosed many years beforehand, that basically just disqualified me straight up. My doctor – I remember at the time the GP was like, "You'd be very lucky to get insurance, if anything." The 200% loading I guess was – and getting a death cover was the only way I could get it, and because it was through my superannuation it was probably the easiest way to get that and probably the cheapest way to get that. But, yeah, it's a shock to get knocked back for something that's unproven and just family history. There was a 50% chance I would have it but, yeah, it's a shock to get knocked back from that especially when it does say specifically Huntington's disease. It's pretty – I think it was pretty discriminatory because there's thousands of conditions out there.

¶59: **Q:** Yeah. No, it would – it does sound like it would be a shock to see something so personally related to you written in a single line, yeah.

¶60: **A:** Yeah, and there weren't many. I remember there weren't many options but that one was just like – I think they call it Huntington's chorea which was pretty outdated as well, which is kind of like, okay, it's not really the term we use now but -

- Uncertain of getting cover
- Need a guarntee
- Needs to be legislated
- Hope that the moratroium will help others
- Insurers being unaware of the condition
- Many people do not have the privilege to be aware of their rights
- Genetic testing can break cycle of disease
- GD in insurance is not really talked about
- Informed consent is important
- Word of mouth
- Finanical limits
- Insurance rejection adds stress to the process
- Impact of Moratorium
- You don't know where the trigger point where I can't get insurance anymore
- Some cover is better than no cover
- I felt that the genetic counselling was pure science.
- Awareness of potential GD
- I don't have disease. It's pretty cut and dry"
- Awareness through support groups
- Using genetic test results
- Challenging the insurance

Coding Density

- A shock to get knocked back

¶61: **Q:** Okay. Yep. In that application did they ask about genetic testing at all from your memory?

¶62: **A:** I think they may have asked if I'd had genetic testing. I can go back to the forms. I'm sure that dossier has the actual form that I filled out as well. I'm pretty sure it's got the whole thing. It's like a big chunky thing. But, yeah – I'm pretty sure I sent it to Jane but I just can't see where it is.

¶63: **Q:** No, that's okay. If she's got it that's really helpful.

¶64: **A:** Yeah. And I just got in touch with Dr Paul Lacaze

¶65: **Q:** Yep. Yeah.

¶66: **A:** Do you know – yep. And Jane at the same time, yeah.

¶67: **Q:** Oh great. You've been in contact with all the important people in the project.

¶68: **A:** Yeah. I mean, that was back I think in 2018, I'm looking at my email there. That was – so I said I came across the articles in their submission to the parliamentary inquiry. I don't know where I found it but it was on LinkedIn or something maybe.

¶69: **Q:** Yep. Cool. It sounds like you're very proactive.

¶70: **A:** Well, I was kind of in the thick of it at that time as well and I was going through the – I was probably going through the court battle. Well, just had gone through that with the company.

¶71: **Q:** Yep.

¶72: **A:** But, yeah, I will definitely send you the dossier as well, whenever I can find it.

¶73: **Q:** Okay. Yeah. Do you remember what the life insurance – did they give you a reason when they rejected the other two insurances, just -

¶74: **A:** Okay. I've got the doc here. It just says, "Unfortunately your application for TPD cover has been declined due to your family history of Huntington's. Unfortunately your application for additional IP cover has been declined due to your family history of Huntington's." This is the death cover, "A premium loading will apply to three units of your death cover due to your family history of Huntington's. The premium loading will be 200% above the premium that will be otherwise charged for a member of your age."

¶75: **Q:** Okay. So it was pretty short. Yep.

¶76: **A:** [unclear] 00:18:00.

¶77: **Q:** Pretty short and simple. Okay.

- Uncertain of getting cover
- Need a guarntee
- Needs to be legislated
- Hope that the moratroium will help others
- Insurers being unaware of the condition
- Many people do not have the privilege to be aware of their rights
- Genetic testing can break cycle of disease
- GD in insurance is not really talked about
- Informed consent is important
- Word of mouth
- A shock to get knocked back
- Finanical limits
- Insurance rejection adds stress to the process
- Impact of Moratorium
- You don't know where the trigger point where I can't get insurance anymore
- Some cover is better than no cover
- I felt that the genetic counselling was pure science.
- Awareness of potential GD
- I don't have disease. It's pretty cut and dry"
- Awareness through support groups
- Using genetic test results
- Challenging the insurance

Coding Density

¶78: **A:** Yep. Yep. That was in 2015, so we go back to 2015.

¶79: **Q:** Yep. Can you – you've then taken this to the complaint's tribunal.

¶80: **A:** Yep.

¶81: **Q:** Do you remember – I think you've already mentioned it but how it was resolved? So if you – I know you've said, yeah, that it's – they've made you retract the complaint and they back paid you. Could you just go into that in a little bit more detail?

¶82: **A:** Yep. Yep. We went to – so I lodged that in 2016 and then we went through basically mediation. We had – it was a teleconference call and I think it was TAL who was the underwriter, so they were the ones who I was up against I guess. They had a few people from their side, maybe three or four people, and then there was someone from the tribunal who was mediating, and then myself. It was basically just sort of to tell my story, they tell their story and then we just met in the middle and they just said, "Look, we'll be happy to" – I think they framed it in a way that it was more a miscommunication that because the policy had changed I was then required to complete a new application form, and I suppose a brand new application. Rather than changing the application and removing loading you have to basically cancel one and do the other. But when I was told they just said, "Complete this form to remove the loading." I said, "Well, no, that's just way too many questions that are irrelevant to why I'm trying to get the loading removed. It just states because of Huntington's disease. I'll tell you about the Huntington's disease. I haven't got it. Here's the form from MCRI it's fine. There's no chance I'll get it." They kept asking me about, "Well, we need to assess your comorbidities." I said, "Look, there's no comorbidities in not having something." I mean, are there? You tell me if there are.

¶83: **Q:** I wouldn't say I'm an expert in Huntington's disease, but I don't think so.

¶84: **A:** You literally cannot get it if you don't have the gene. Now, I don't know what the – guilt, is that a comorbidity, if you're not getting it and your family member does? I don't know.

¶85: **Q:** Not really related though, is it?

¶86: **A:** But they ask you so many other questions. Yeah, they just kept asking me, "Oh, we need to assess your comorbidities. Comorbidities." I said, "There's no comorbidities I can see, so I'm not going to tell you about my whole medical history again, because it's irrelevant." So that's – yeah, so I went through the mediation, and then I just wanted it to come to an end and I was like, "Oh fine." Then they said, "We'll backdate it. We'll pay you the money." It was only, as I said, a couple of hundred bucks. But it sort of ended that they would review their processes and take a more sensitive approach to these sorts of things.

¶87: **Q:** Yep. You said -

¶88: **A:** That's how it seemed to me.

- Uncertain of getting cover
- Need a guarntee
- Needs to be legislated
- Hope that the moratroium will help others
- Insurers being unaware of the condition
- Many people do not have the privilege to be aware of their rights
- Genetic testing can break cycle of disease
- GD in insurance is not really talked about
- Informed consent is important
- Word of mouth
- A shock to get knocked back
- Finanical limits
- Insurance rejection adds stress to the process
- Impact of Moratorium
- You don't know where the trigger point where I can't get insurance anymore
- Some cover is better than no cover
- I felt that the genetic counselling was pure science.
- Awareness of potential GD
- I don't have disease. It's pretty cut and dry"
- Awareness through support groups
- Using genetic test results
- Challenging the insurance

Coding Density

¶89: **Q:** Yeah. You said you were telling your story and they were telling their story. Was their story just the needing to know your comorbidities or what was their story so to speak?

¶90: **A:** Yeah. The story, as far as I can remember, was basically this was our process, we need to reassess. It was more like a procedural sort of thing. "Have to create a new policy. We can't just amend it. The existing policy it needs to be done from the beginning. What's why we need to ask these questions." I was fine with that. I was like, "Okay. This is three or four years down the track. I don't really care that much anymore but I just want to see this come to an end and if it's" – I don't want to go to an official court about this. I think it's come far enough. I'm happy to just do the form which I did. I did the form in the end and they back paid it and it was just okay, let's move on. But it was more – look, I wanted them to know how much of an illogical process it was or communicated wrong or something. I don't know. I mean, it was just – it just seemed like they hadn't really thought it through. They're asking me to – and it just seemed like a template response to anything. Hopefully they've changed. I don't know.

¶91: **Q:** I don't know. I think they're all a bit different.

¶92: **A:** Yeah. Yeah.

¶93: **Q:** What about with the income protection and the total permanent disability, did you ever reapply for those?

¶94: **A:** Yeah, I'm pretty sure. You made me think about that. I'm 100% sure I did, and if I didn't I would do it now but I'm pretty sure I just topped it right up when I reapplied, yeah.

¶95: **Q:** I mean, from memory you didn't have any issues the second time round with those?

¶96: **A:** No. None, at all, yeah. Yep. I will check about that straight after this call. I'm pretty sure I did. Yep.

¶97: **Q:** Yeah. Okay.

¶98: **A:** But, yeah, no, I don't recall having any issues at all, and I think they wouldn't give me any issues to be honest. We've come a long way together.

¶99: **Q:** You've been through a lot with them. Is there anything on that topic on your personal experiences there that you think would be helpful for us to know that you haven't already mentioned?

¶100: **A:** No. I'm sorry, this sort of seems like I'm complaining a lot about that one particular aspect but that was the whole journey from my point of view. I mean, it was rejected straight up, 200% premium, I had cover up to 2016. Going through the testing, you don't know what's going to be the outcome. If it had have been a positive result then kind of restricted in a lot of ways and having seen my dad go through his decline, it's pretty full on to not have the protection that someone else would have. There's a lot going on.

- Uncertain of getting cover
- Need a guarntee
- Needs to be legislated
- Hope that the moratroium will help others
- Insurers being unaware of the condition
- Many people do not have the privilege to be aware of their rights
- Genetic testing can break cycle of disease
- GD in insurance is not really talked about
- Informed consent is important
- Word of mouth
- A shock to get knocked back
- Finanical limits
- Impact of Moratorium
- Insurance rejection a
- You don't know where the trigger point where I can't get insurance anymore
- Some cover is better than no cover
- I felt that the genetic counselling was pure science.
- Awareness of potential GD
- I don't have disease. It's pretty cut and dry"
- Awareness through support groups
- Using genetic test results
- Challenging the insurance

Coding Density

¶101: **Q:** Yeah. No, it's definitely not something to apologise about complaining for. It's worthy of complaint.

¶102: **A:** No, no. Yeah. Look, I mean, I hope other people don't have to have that – go through that and have that uncertainty and everything and hopefully the moratorium will give people a bit more flexibility and a bit more cover because if I had have been positive and then started being symptomatic now, I mean, that's – it's full on. And plus you – probably most people in the situation with the thing about having kids as well and it's just such an expensive thing if you go down IVF and everything and then you're not protected, and if you're positive it's just – it's full on. It's full on. So hopefully the moratorium does give people more security, at least perceived security, for their journey. But, yeah, look I will send that document. I mean, it's huge. Feel free to use it. I don't know if it's – there's a confidentiality aspect to it. I didn't see that but it's mine so I guess I can give it. I didn't sign anything to say this is a confidentiality agreement, so I'm happy for you to use it and with your research team have a look at it and see if there's anything useful.

¶103: **Q:** Yeah, awesome. Thank you.

¶104: **A:** There may not be. But it does give it a timeline.

¶105: **Q:** Yep. I know we started a little bit late. How are you going for time? Are you happy to answer a few more questions or?

¶106: **A:** Yeah, yeah, yep, yep. Yep, I will. Yep.

¶107: **Q:** I'll just ask you some general questions now about your opinion on the moratorium and if you can relate it back to your own experiences, that's great.

¶108: **A:** Yep.

¶109: **Q:** You've obviously said that you don't think life insurance companies should be use applicant's genetic test results to decline or restrict cover. Can you tell me why you feel that way?

¶110: **A:** It just feels so discriminatory. I mean, especially if there's no guarantee that the person will inherit such diseases and so on and it's just creates such uncertainty for a lot of people. It's another level of stress. I know that these companies are businesses and they have to protect their own risk and have to take on risk and so on. Whether it is unlimited, I think I said unlimited is probably the way to go but, I mean, it may not be achievable for these companies to even operate. Having some cover is better than no cover. As I got a little bit of cover with a premium but then got rejected for others, I think there should be some sort of level of some cover at least.

¶111: **Q:** Yeah.

¶112: **A:** I mean, it's a hard question. It's a very difficult question because if these companies don't exist then we're not covered for anything if they can't take on the risk.

- Uncertain of getting cover
- Need a guarntee
- Needs to be legislated
- Hope that the moratorium will help others
- Insurers being unaware of the condition
- Many people do not have the privilege to be aware of their rights
- Genetic testing can break cycle of disease
- GD in insurance is not really talked about
- Informed consent is important
- Word of mouth
- A shock to get knocked back
- Finanical limits
- Insurance rejection adds stress to the process
- Impact of Moratorium
- You don't know where the trigger point where I can't get insurance anymore
- Some cover is better than no cover
- I felt that the genetic counselling was pure science.
- Awareness of potential GD
- I don't have disease. It's pretty cut and dry"
- Awareness through support groups
- Using genetic test results
- Challenging the insurance

Coding Density

¶113: **Q:** Yeah. Is there a specific exception that you could think of where you think maybe not?

¶114: **A:** I mean, it's difficult. I mean, having – being explicit about a particular disease, from my point of view on the form when it said Huntington's disease, I mean, I don't think it should be spelt out like that. I don't think it should be straight out disease focused like that. It's a difficult question. That's a very difficult question. I mean, the majority of me says no, they shouldn't use that but then a little part of me says well, how can they balance their own risk? It is difficult. My short answer is no, they shouldn't use it.

¶115: **Q:** Okay. That's a nice summary.

¶116: **A:** But it's complicated. But it's complicated.

¶117: **Q:** Yeah. Awesome. You've also indicated that you think it's negative, that the moratorium is currently not permanent and that in 2024 it'll expire if you don't renew it.

¶118: **A:** Yep.

¶119: **Q:** Can you tell me why it's a negative?

¶120: **A:** Because there's no guarantee it will continue and then – I mean, these are businesses. Who knows what – it's not a Government running this. I mean – is that right? I mean -

¶121: **Q:** Yeah.

¶122: **A:** - it's up to the companies themselves to kind of sign up to this. I mean, you'd want some sort of guarantee and the world's pretty crazy right now. Who knows what's going to happen and what the position is, so you'd want it to be a permanent agreement.

¶123: **Q:** Yeah. That was also one of the questions. It's currently not legislation that's managed by the Australian Government but you've indicated that it should be. Do you want to elaborate a little bit on that?

¶124: **A:** Yeah. I mean, the Government you would hope would have the greater good in mind and the public good rather than – from the point of view of a company with shareholders. I think it needs to be legislated so it's – so everybody has access and that's not just at the whim of companies to keep these agreements in place, because things change at the company level, but once it's in legislation – I mean, there are checks and balances about that to make it more permanent and more – for the public good really.

¶125: **Q:** Yeah. So it's about permanency and more of a public trust I guess, in the -

¶126: **A:** Absolutely, yeah, yeah.

¶127: **Q:** Yeah. Do you think that would have an impact on people's decisions to have genetic testing, whether it's permanent or government-run?

- Uncertain of getting cover
- Need a guarntee
- Needs to be legislated
- Hope that the moratroium will help others
- Insurers being unaware of the condition
- Many people do not have the privilege to be aware of their rights
- Genetic testing can break cycle of disease
- GD in insurance is not really talked about
- Informed consent is important
- Word of mouth
- A shock to get knocked back
- Finanical limits
- Insurance rejection adds stress to the process
- Impact of Moratorium
- You don't know where the trigger point where I can't get insurance anymore
- Some cover is better than no cover
- I felt that the genetic counselling was pure science.
- Awareness of potential GD
- I don't have disease. It's pretty cut and dry"
- Awareness through support groups
- Using genetic test results
- Challenging the insurance

Coding Density

¶128: **A:** Yeah, yeah, absolutely. Absolutely.

¶129: **Q:** Yep.

¶130: **A:** Absolutely. I mean, if there was such – even going through this with my cousin recently. Her mum tested positive. It was my auntie, so my cousin's going through – basically me in 2016 or '15 or whatever. I don't think anything's really changed in terms of the kind of – lack of a better word, the black market, of "what do I do". I don't know what to do. Who can tell me what to do? And even – it seems like it's – we're all going through it for the first time, which we are, but there's no certainty of will I get cover, will I not get cover? I don't know. Who knows? It's quite difficult. And then another cousin comes along and the same story, "Where do I go? What do I do?", and then we become just sort of sharing, "Well, this is what I did. This is what I did." There's no sort of like certainty, yep, get the test. No. We can break the cycle of this disease if we all know if we've got it or not and not choose to have kids in a certain way or – it doesn't make sense not to have the test because you're worried about the insurance side of it.

¶131: **Q:** Yep. Yeah, no, that was very interesting aspect of it from my perspective. What you went through is that you were hearing it from your family members and support groups instead of anyone official, yeah.

¶132: **A:** Yeah. But, I mean, where would I go for that anyway? I mean, is there a place that can – you can go to find that information out? I don't know.

¶133: **Q:** It's hard. Yeah. I think genetic counsellors before you go for your test are supposed to talk about it.

¶134: **A:** I went through genetic counselling but that was more – and that was at MCRI or VCGS and I felt that to be more scientific than the kind of social aspects of it. That's where I put the brakes on to be honest. I put the brakes on and halted the process a bit and just said, "No, I need to get stuff sorted out", you know, the family counselling and then there's financial counselling because I felt that the genetic counselling was pure science. This is how it works, alleles and all that sort of stuff rather than the kind of social aspect of it. I don't know if that's a thing that the VCGS should be taking on. Would they want to be advising on that? Maybe they wouldn't. I don't know. It's not really [unclear] 00:33:11.

¶135: **Q:** Yeah. I'm not sure how much they say or if it's just supposed to be like a little disclaimer. I'm not 100% sure but it is something we're trying to figure out is how to let people know that this is even a thing, which is why we ask about it.

¶136: **A:** Well, it's going to – they're putting so much money into this as well at the Government level. You want people to be taking up these tests. I mean, the Mackenzie's Mission stuff and everything and people are starting to have genomic testing as part of routine practice. I mean, you'd want people to be taking it up and knowing what the risks are or not of starting the process. Because once you've started the process, you're on the conveyor belt. You've had the blood test and everything, you're kind of on the way.

- Need a guarntee
- Uncertain of getting cover
- Needs to be legislated
- Hope that the moratroium will help others
- Insurers being unaware of the condition
- Many people do not have the privilege to be aware of their rights
- Genetic testing can break cycle of disease
- GD in insurance is not really talked about
- Informed consent is important
- Word of mouth
- A shock to get knocked back
- Finanical limits
- Insurance rejection adds stress to the process
- Impact of Moratorium
- You don't know where the trigger point where I can't get insurance anymore
- Some cover is better than no cover
- I felt that the genetic counselling was pure science.
- Awareness of potential GD
- I don't have disease. It's pretty cut and dry"
- Awareness through support groups
- Using genetic test results
- Challenging the insurance

Coding Density

¶137: **Q:** Yeah. Yeah, no, very interesting.

¶138: **A:** Yeah.

¶139: **Q:** We've just touched on the last question I had which was about people being aware of it and how do we think that can be improved. Yeah. Is there anything else you think I should know?

¶140: **A:** I guess, yeah – I don't know. I mean, I'm only really involved with that support group because my mother was involved with this through my dad and – but I would say the majority of people aren't involved with support groups. Or if there's a first time that there is a diagnosis in a family of a genetic disease and it's got to start somewhere, someone's got to find out about it for the first time, where do those people go? I don't know. If they're part of a church or whatever, they go to that counselling and they're not going to know. It's hard to communicate. A lot of these people with genetic diseases, especially with Huntington's, they come from not very socio-economically privileged backgrounds or anything because of the – it's the nature of the disease. There's a whole demographic of people who probably don't know where to go at all, not really literate in this sort of space. I think we were lucky that we had people that could navigate it but I would say the majority don't. That's a tough one. I don't know, do you get anything with Centrelink or something? I don't know. Through Medicare? I don't know. So it kind of covers everyone.

¶141: **Q:** Yeah. Yeah, I'm not sure.

¶142: **A:** I don't know the answer to that. Tough problem.

¶143: **Q:** Yeah, it is, isn't it?

¶144: **A:** Yeah. It's a scary one because once you start getting on the journey you don't know is this the trigger point where I can't get insurance anymore? Have I gone too far even just asking a question? I don't even know if I want it on my GP's record that I'm considering it. That was a question I remember having, how far is too far? That's why I wanted to get the insurance done straight up. But then there was that catch where I – they did ask about Huntington's so I can't lie on that. So that's where that stopped for me but, yeah, I don't know. Tough one.

¶145: **Q:** Yeah. Well, thank you so much for your time. Is there anything generally about anything we've covered on that you want to add?

¶146: **A:** Not really, no. I mean, yeah, it's complicated.

¶147: **Q:** It really is, yeah.

¶148: **A:** Yeah. But thanks for taking on this role. I mean, if you're not doing it then no one's doing it so it's helping a lot of people. And because it's becoming more routine, I think anyway from seeing stuff in the news, it's going to help a lot of people just having that certainty and not have that kind of doubt. You don't want people to not go through some sort

- Uncertain of getting cover
- Need a guarntee
- Needs to be legislated
- Hope that the moratroium will help others
- Insurers being unaware of the condition
- Genetic testing can break cycle of disease
- GD in insurance is not really talked about
- Informed consent is important
- Word of mouth
- A shock to get knocked back
- Finanical limits
- Insurance rejection adds stress to the process
- Impact of Moratorium
- Some cover is better than no cover
- I felt that the genetic counselling was pure science.
- Awareness of potential GD
- I don't have disease. It's pretty cut and dry"
- Awareness through support groups
- Using genetic test results
- Challenging the insurance
- Many people do not have the privilege to be aware of their rights
- You don't know where the trigger point where I can't get insurance anymore

Coding Density

of medical procedure because there's some doubt about finances or something. It's not worth it.

¶149: **Q:** Yeah, absolutely. Yeah, thank you for that. That's very kind.

¶150: **A:** I'm trying to find – if I didn't send that I will send it.

¶151: **Q:** Yep.

¶152: **A:** I'll get in touch about where I can send it because it's quite a big document. I might not have – I'm just looking. I can't see where – that I have sent it. But I think I just said to Jane that I have got it available if you want it.

¶153: **Q:** Okay.

¶154: **A:** But if you do want it I'll send it and away we go.

¶155: **Q:** Yeah, perfect. So as well I will be obviously taking notes from everything we've talked about today, so if I want something clarified or anything like that, are you happy for me to reach out to you again?

¶156: **A:** Yeah, yeah, no problem. Yeah, yep, I'm right. Yep.

¶157: **Q:** Okay. Thank you.

- Uncertain of getting cover
- Need a guarntee
- Needs to be legislated
- Hope that the moratroium will help others
- Insurers being unaware of the condition
- Many people do not have the privilege to be aware of their rights
- Genetic testing can break cycle of disease
- GD in insurance is not really talked about
- Informed consent is important
- Word of mouth
- A shock to get knocked back
- Finanical limits
- Insurance rejection adds stress to the process
- Impact of Moratorium
- You don't know where the trigger point where I can't get insurance anymore
- Some cover is better than no cover
- I felt that the genetic counselling was pure science.
- Awareness of potential GD
- I don't have disease. It's pretty cut and dry"
- Awareness through support groups
- Using genetic test results
- Challenging the insurance

Coding Density
